# The Assembly of Bacteria Living in Natural Environments Shapes Neuronal Integrity and Behavioral Outputs in Caenorhabditis elegans

**DOI:** 10.1128/mbio.03402-22

**Published:** 2023-03-08

**Authors:** Sebastian Urquiza-Zurich, Victor Antonio Garcia-Angulo, Paula Burdisso, M. Fernanda Palominos, Lucia Fernandez-Hubeid, Paloma A. Harcha, Juan P. Castillo, Andrea Calixto

**Affiliations:** a Centro Interdisciplinario de Neurociencia de Valparaíso, Facultad de Ciencias, Universidad de Valparaíso, Valparaíso, Chile; b Instituto de Ciencias Biomédicas, Facultad de Medicina, Universidad de Chile, Santiago de Chile, Chile; c Instituto de Biología Molecular y Celular de Rosario (IBR-CONICET), Facultad de Ciencias Bioquímicas y Farmacéuticas, Universidad Nacional de Rosario, Rosario, Santa Fe, Argentina; d Plataforma Argentina de Biología Estructural y Metabolómica (PLABEM), Rosario, Santa Fe, Argentina; e Facultad de Ciencias Químicas, Universidad Nacional de Córdoba, Córdoba, Argentina; f Instituto de Farmacología Experimental (IFEC), CONICET, Córdoba, Argentina; University of Texas Health Science Center at Houston

**Keywords:** life-history traits, metabolomics, nematode, neurodegeneration, wild bacteria

## Abstract

Bacterivore nematodes are the most abundant animals in the biosphere, largely contributing to global biogeochemistry. Thus, the effects of environmental microbes on the nematodes’ life-history traits are likely to contribute to the general health of the biosphere. Caenorhabditis elegans is an excellent model to study the behavioral and physiological outputs of microbial diets. However, the effects of complex natural bacterial assemblies have only recently been reported, as most studies have been carried out with monoxenic cultures of laboratory-reared bacteria. Here, we quantified the physiological, phenotypic, and behavioral traits of C. elegans feeding on two bacteria that were coisolated with wild nematodes from a soil sample. These bacteria were identified as a putative novel species of *Stenotrophomonas* named *Stenotrophomonas* sp. strain Iso1 and a strain of Bacillus pumilus designated Iso2. The distinctive behaviors and developmental patterns observed in animals fed with individual isolates changed when bacteria were mixed. We studied in more depth the degeneration rate of the touch circuit of C. elegans and show that B. pumilus alone is protective, while the mix with *Stenotrophomonas* sp. is degenerative. The analysis of the metabolite contents of each isolate and their combination identified NAD^+^ as being potentially neuroprotective. *In vivo* supplementation shows that NAD^+^ restores neuroprotection to the mixes and also to individual nonprotective bacteria. Our results highlight the distinctive physiological effects of bacteria resembling native diets in a multicomponent scenario rather than using single isolates on nematodes.

## INTRODUCTION

All eukaryotes live intimately with microbial communities. In their interaction, communication with microbes substantially impacts the life-history traits of the eukaryotic hosts (1, 2). However, how the native microbiota affects the life-history traits of the host and promotes adaptation is still largely unknown. The bacterivore nematode Caenorhabditis elegans offers a simple framework to manipulate the composition and metabolite content of its microbial diet to dissect specific molecules that direct behavior and phenotypic outputs in the animal.

C. elegans has typically been grown on monoxenic cultures of Escherichia coli strain OP50 (3). Feeding worms on other bacteria allowed the discovery that the developmental rate depends on specific bacterial metabolites such as vitamin B_12_ (4) or that neurodegeneration can be delayed by γ-aminobutyric acid (GABA) produced by E. coli HT115 (5). Those pioneering works, however, were also done with monocultures of laboratory bacteria, opening the question of how the complex natural microbiota influences behavior and physiology. We hypothesize that coexisting consortia of environmental bacteria found in the wild promote behavioral signatures that differ from the behaviors observed in C. elegans fed on a single laboratory bacterial species. The study of environmental bacteria might also serve as a platform to dissect the benefits or detriments that microbial associations can have on hosts in natural environments.

The microbiota of C. elegans and other Rhabditida species from Europe and North America is composed mainly of species of the *Enterobacteriaceae* family (6) and the Pseudomonas, *Stenotrophomonas*, *Xanthomonas*, *Ochrobactrum*, and *Sphingomonas* genera (7, 8). However, nematodes from South America and their associated bacteria have not been studied. We isolated bacteria carried by wild nematodes from the soil in a semiarid location in central Chile, identifying a potentially new species of *Stenotrophomonas* and a novel strain of Bacillus pumilus. We found that monocultures produced different life-history trait outputs than cocultures in worms. B. pumilus acts as a neuroprotector in C. elegans with a degenerating touch circuitry, while its coculture with *Stenotrophomonas* eliminates this adaptive trait. Using metabolomic analysis of single bacterial cultures compared to their mix coupled with *in vivo* validation, we identified bacterial NAD^+^ as a neuroprotective metabolite.

## RESULTS

### Identification of microbes coisolated with wild Chilean nematodes.

Adaptation to environmental change requires the work of coinhabiting organisms, especially those that are most ubiquitous, such as bacteria and nematodes. To reveal how bacteria in natural environments modulate the life-history traits of the nematode C. elegans, we collected nematodes from a Chilean scrubland characterized by mountain ranges and Andean steppes with increasingly prominent hydric stress. We placed soil samples onto growth plates seeded with E. coli OP50 lawns, the standard laboratory diet of C. elegans. Wild worms present in the soil sample migrated toward E. coli OP50-carrying bacteria that grew on the plates. Wild nematodes preferred to feed on these colonies for generations rather than on E. coli OP50. We isolated two distinctive colonies from the wild bacterial growth (see Materials and Methods). These isolates were named Iso1 and Iso2.

**FIG 1:**
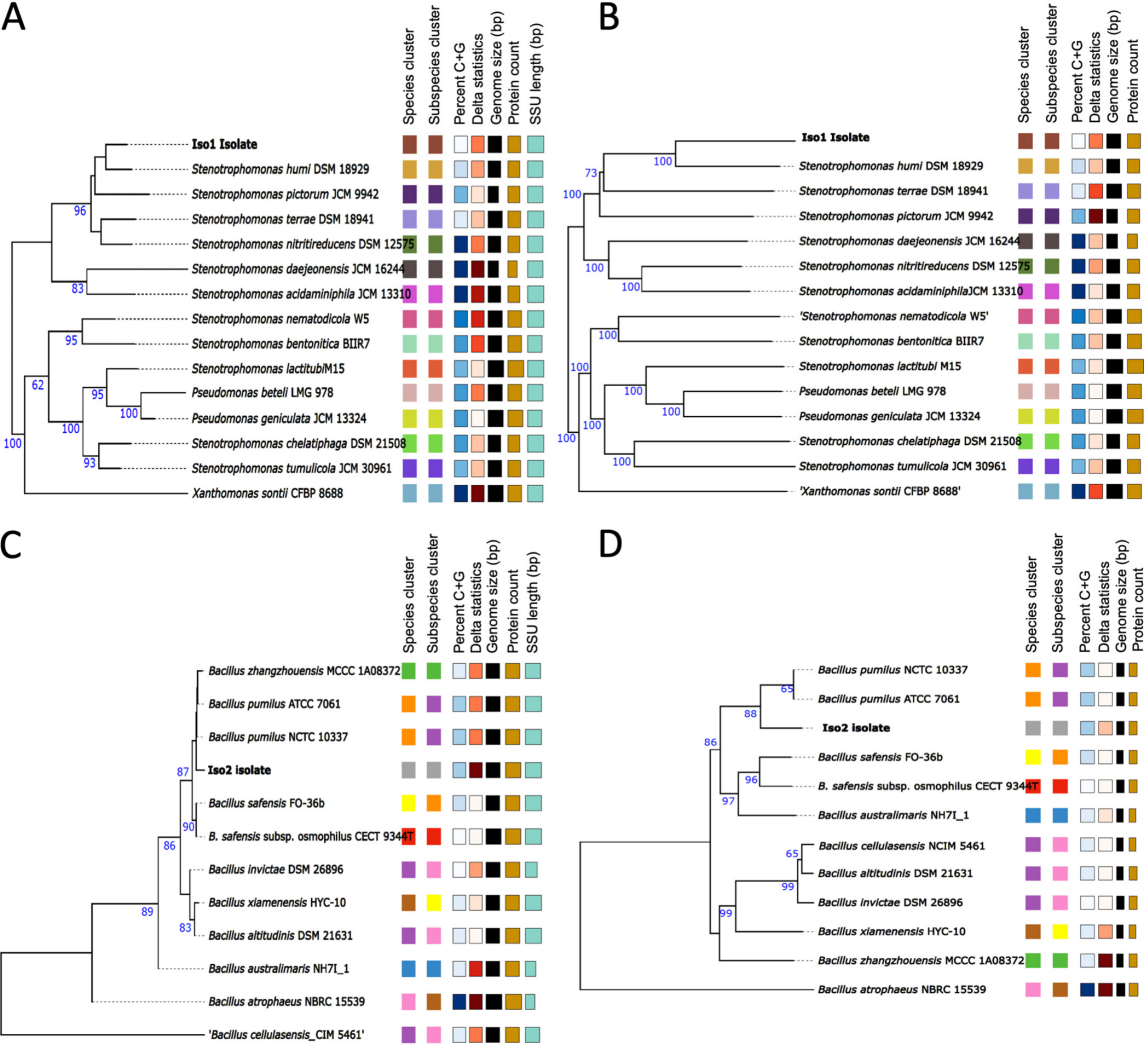
TYGS phylogram results for the bacterial isolates. Shown are a 16S-based phylogram (A and C) and a GBDP-based phylogram (B and D) for Iso1 and Iso2, respectively. In both cases, the phylogram analysis includes the closest type strains in the databases. The colors of the squares group strains belonging to the same cluster as defined by each of the parameters indicated at the top of the squares. SSU, small-subunit rRNA.

We sequenced the genomes of Iso1 and Iso2, followed by genome-based identification analysis. We employed the Type Strain Genome Server (TYGS) pipeline for the identification of species (Fig. 1). This was complemented by the determination of the average nucleotide identity (ANI) between the isolate genomes and their closest type strains detected by TYGS using OrthoANIu (9). For Iso1, the closest type strain was Stenotrophomonas humi DSM 18929. Nonetheless, both the 16S-based and the genome BLAST distance phylogeny (GBDP)-based phylograms (Fig. 1A and B) locate the isolate in different species and subspecies clusters than those of this type strain. Digital DNA-DNA hybridization (dDDH) analysis renders a *d*_4_ value of 37.8% of digital hybridization with the genome of S. humi DSM 18929. With the dDDH procedure, a *d*_4_ value of <70% indicates separate species (10) (Table 1). The 37.8% dDDH value with a confidence interval (CI) of 35.4 to 40.3% for the comparison against *S. humi* DSM 18929 places the isolate well beyond the threshold to be considered a new species. Moreover, OrthoANIu analysis determined an ANI of 89.5% for the comparison of Iso1 against *S. humi* DSM 18929 (an ANI value of >95 to 97% indicates that strains belong to the same species). Thus, even using the loose 95% ANI threshold, this result supports that Iso1 is a novel species within the *Stenotrophomonas* genus. Hence, this isolate was named *Stenotrophomonas* sp. strain Iso1.

**TABLE 1:** dDDH analysis results

Isolate	Closest type strain	% dDDH (*d*_4_ value)	*d*_4_ CI (%)	OrthoANIu value (%)
Stenotrophomonas sp. Iso2	Stenotrophomonas humi DSM 18929	37.8	35.3–40.3	89.51
Bacillus sp. Iso3	Bacillus pumilus NCTC 10337	63.1	60.2–65.9	95.54

The 16S-based and GBDP-based phylograms (Fig. 1C and D) place Iso2 in different species and subspecies clusters than those of its closest type strain, Bacillus pumilus NCTC 10337. Nonetheless, the dDDH value obtained against this type strain is 63.1%, with a CI of 60.2 to 65.9% (Table 1). These values are close to the 70% threshold. Moreover, the OrthoANIu value of Iso2 against this species is 95.54%, which indicates that the isolate is a strain of *B. pumilus*. Thus, it is not clear whether this bacterium represents a novel species separated from *B. pumilus*; hence, we designated the isolate Bacillus pumilus Iso2.

While previous studies of bacterium-worm associations in South America are lacking, there is evidence that *Stenotrophomonas* strains are part of the natural microbiota of C. elegans (11). *B. pumilus*, however, is a ubiquitous soil microbe associated with plants and environmental surfaces (12–14).

### C. elegans dietary choice of natural isolates and their consortia is bacterial context dependent.

**FIG 2:**
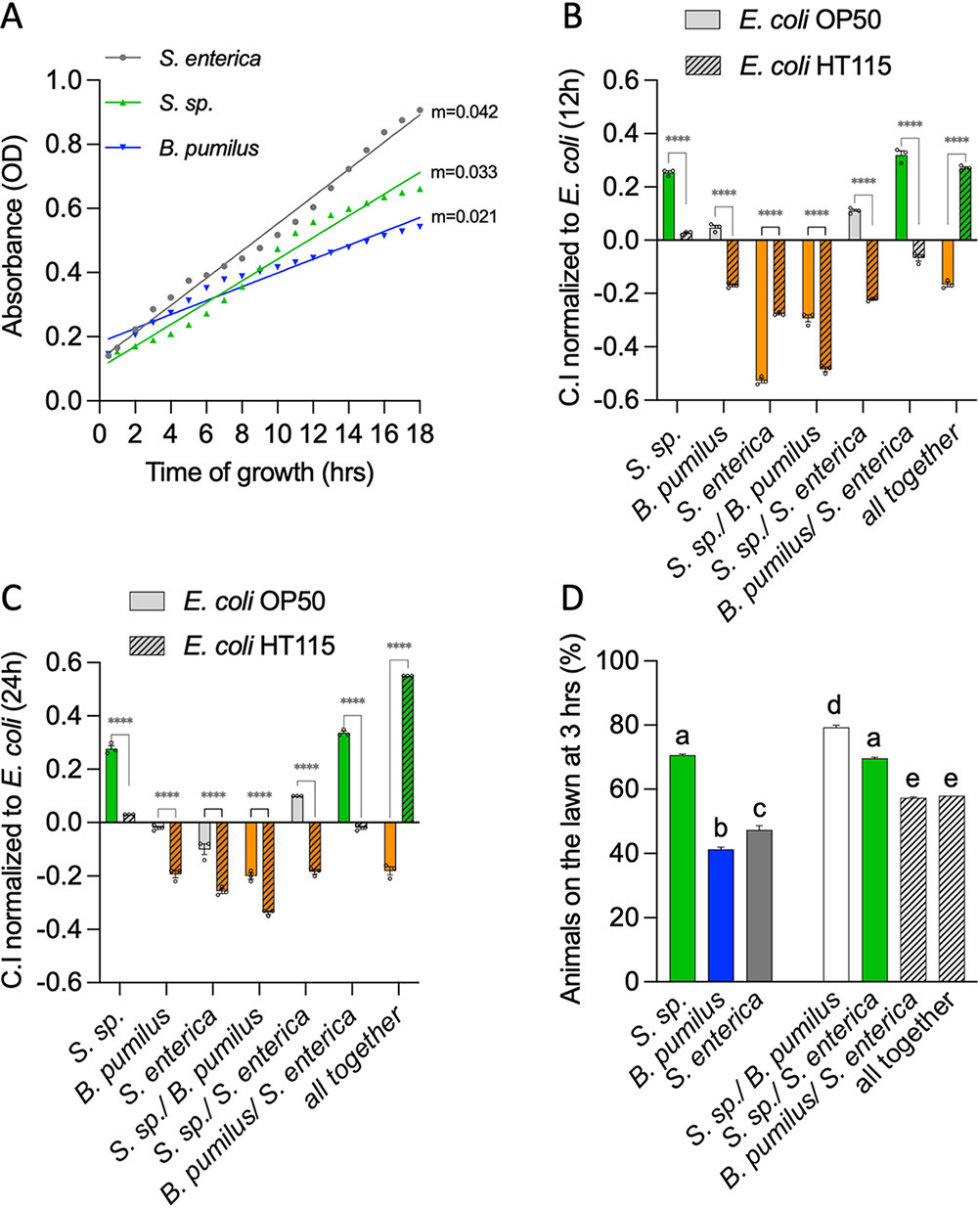
C. elegans preference for bacteria and consortia. (A) Growth of individual bacteria in liquid culture over time. m indicates the value of the slope on a simple linear regression of the curves shown with symbols for each bacterium. (B and C) Chemotaxis index [(no. of worms on test bacteria − no. of worms on the E. coli control)/total no. of animals] for each bacterium or mixes normalized to E. coli OP50 (B) or E. coli HT115 (C) 12 and 24 h after exposure to the food choice (*n* = 3) (two-way ANOVA). (D) Percentage of animals inside individual bacterial lawns or mixes 3 h after placing them in the center of the lawn (*n* = 3) (ordinary one-way ANOVA). The same letter indicates no significant differences; different letters indicate significant differences. ****, *P* < 0.0001; ***, *P* < 0.001; **, *P* < 0.005; *, *P* < 0.05. The underlying numerical data and statistical analysis for each figure panel can be found in S1 and S2 Datasets, respectively.

*Stenotrophomonas* sp. Iso1 and *B. pumilus* Iso2 are likely part of the natural diet of wild nematodes. We tested the preference of laboratory-reared C. elegans nematodes for the isolates, individually or in consortia, compared to E. coli OP50 or E. coli HT115 at 12 and 24 h. The test also included Salmonella enterica, which can infect and cause disease in worms, thus representing a detrimental diet (15). The three bacteria grow at similar rates, with S. enterica having a slightly higher growth rate than the other isolates (Fig. 2A).

The nematodes’ choice of bacterial food depended on the strain of E. coli offered as a counterpart. *Stenotrophomonas* sp. was preferred over E. coli OP50 but was equally as selected as E. coli HT115. Animals chose *B. pumilus* and E. coli OP50 similarly, while HT115 was preferred (Fig. 2B and C). S. enterica, in contrast, caused worms to strongly prefer E. coli OP50 or HT115 (Fig. 2B). This preference became smaller at 24 h for E. coli OP50 (Fig. 2C).

When bacteria were mixed, animals preferred either E. coli strain over the combination of the two wild isolates. The mixes of either wild isolate with S. enterica were preferred over OP50 but not HT115 (Fig. 2B and C). Contrary to the experiment with E. coli OP50, the mix of the three bacteria was markedly preferred over E. coli HT115 (Fig. 2B and C). These results indicate that food choice is context dependent and that in addition to novelty versus familiarity (16), worms might sense molecules present only in the cocultures that direct their preference. Also, with the exception of S. enterica, most choices were maintained at 24 h.

We next examined the tendency of animals to remain in lawns or to migrate outside after 1, 2, and 3 h. The lawn with the highest occupancy was *Stenotrophomonas* sp., with 70% at 3 h (Fig. 2D), while S. enterica and *B. pumilus* retained <50% occupancy (the complete quantification is shown in Fig. S1 in the supplemental material). The mix of the two wild isolates retained over 80% of the animals, indicating that when not competing with E. coli OP50, their mix is highly liked. The mix of S. enterica with *B. pumilus* or the two wild isolates resulted in higher occupancies than those with S. enterica or *B. pumilus* alone. In this experiment, mixing S. enterica with *Stenotrophomonas* sp. did not affect the high worm retention rate produced by *Stenotrophomonas* sp.

10.1128/mbio.03402-22.1 FIG S1 Percentages of animals inside, on the border of, and outside individual bacterial lawns or mixes 1, 2, and 3 h after placing them in the center of the plate. Download FIG S1, TIF file, 2.6 MB. Copyright © 2023 Urquiza-Zurich et al. 2023 Urquiza-Zurich et al. https://creativecommons.org/licenses/by/4.0/ This content is distributed under the terms of the Creative Commons Attribution 4.0 International license.

10.1128/mbio.03402-22.2 FIG S2 Percentages of animals in larval or adult stages or only adults 24 h (A), 48 h (B), and 72 h (C) after exposure to single bacteria or consortia. Download FIG S2, TIF file, 2.6 MB. Copyright © 2023 Urquiza-Zurich et al. 2023 Urquiza-Zurich et al. https://creativecommons.org/licenses/by/4.0/ This content is distributed under the terms of the Creative Commons Attribution 4.0 International license.

We next asked whether nematodes dwell or roam on bacterial lawns. Animals dwelled on E. coli HT115 and roamed on E. coli OP50, as reported previously for good and mediocre diets, respectively (17). Animals dwelled on *B. pumilus* but roamed on *Stenotrophomonas* sp. and S. enterica (Table 2). The mix of *B. pumilus* and *Stenotrophomonas* sp. caused dwelling. The mix of S. enterica with *B. pumilus* or *B. pumilus-Stenotrophomonas* sp. switches animals to roaming, suggesting that the locomotion pattern of the animals is sensitive to changes in bacterial composition.

**TABLE 2:** Behavior of nematodes on bacterial lawns

Bacterial treatment	Presence of behavior in the bacterial lawn	
	Dwelling	Roaming
E. coli OP50		•
E. coli HT115	•	
S. enterica		•
*Stenotrophomonas* sp.		•
*B. pumilus*	•	
S. enterica-*Stenotrophomonas* sp.		•
S. enterica-*B. pumilus*		•
*Stenotrophomonas* sp.-*B. pumilus*	•	
All together		•

### *B. pumilus* accelerates pharyngeal pumping and defecation rates.

**FIG 3:**
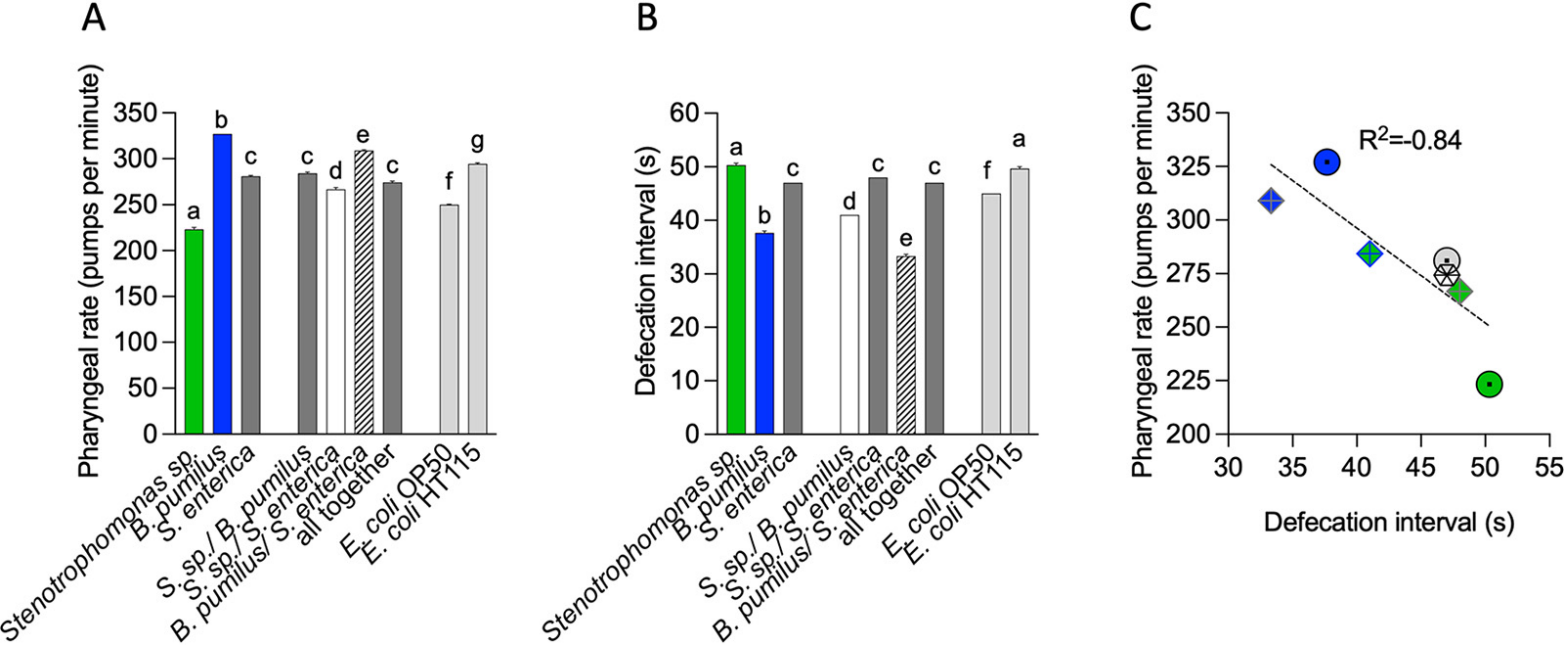
Pharyngeal pumping and defecation rates in individual bacteria and consortia. (A) Pharyngeal pumping rates (pumps per minute) of L4 animals feeding on single bacteria or consortia for one generation. E. coli OP50 and E. coli HT115 served as the controls, which were performed along with the other quantifications (*n* = 3) (one-way ANOVA). (B) Defecation rates (anal contractions per minute) of L4 animals feeding on single bacteria or consortia for one generation. E. coli OP50 and E. coli HT115 served as the controls, which were performed along with the other quantifications (*n* = 3) (one-way ANOVA). (C) Correlation between pharyngeal pumping rates and defecation rates using the Pearson test. Circles indicate individual bacteria (green, *Stenotrophomonas* sp.; blue, *B. pumilus*; gray, S. enterica), rhomboids indicate consortia of two bacteria (green with blue lines, *Stenotrophomonas* sp.-*B. pumilus*; blue with gray lines, *B. pumilus*-S. enterica; green with gray lines, *Stenotrophomonas* sp.-S. enterica), and the diamond represents the mix of all bacteria. The underlying numerical data and statistical analysis for each figure panel can be found in S1 and S2 Datasets, respectively.

As a measure of the feeding rate, we measured the number of contractions of the pharynx per minute (18) and the defecation rate, a rhythm coupled to peristaltic movements caused by feeding. *B. pumilus* generates the highest number of pumps per minute (ppm), with a mean of 327, followed by E. coli HT115 (mean of 293). *Stenotrophomonas* sp. decreased the pharyngeal pumping rate to 223 ppm, the lowest rate observed (Fig. 3A). The ppm produced by the mix of wild isolates resembled the average between the two individual bacteria, as did the mix with S. enterica, suggesting that each bacterium influences the pumping rate equally.

C. elegans nematodes defecate every 45 s on E. coli OP50 (19) (Fig. 3B). *B. pumilus* alone increases the defecation rate to a mean of every 37 s, which is also consistent with the increased pumping rate. The defecation rates for *Stenotrophomonas* sp. (mean, every 50 s) and S. enterica (mean, every 47 s) were similar to those for the E. coli controls (Fig. 3B). *B. pumilus* mixed with S. enterica generated a shorter defecation interval than did *B. pumilus* alone (mean, 33 s) and *Stenotrophomonas* sp. with *B. pumilus* (mean, 41 s), averaged for the individual isolates (Fig. 3B). We correlated the rates of defecation and pharyngeal pumping using a Pearson test (Fig. 3C) and found an inverse correlation where accelerated pumping implies a shorter defecation time.

### Wild isolates delay development into adults.

**FIG 4:**
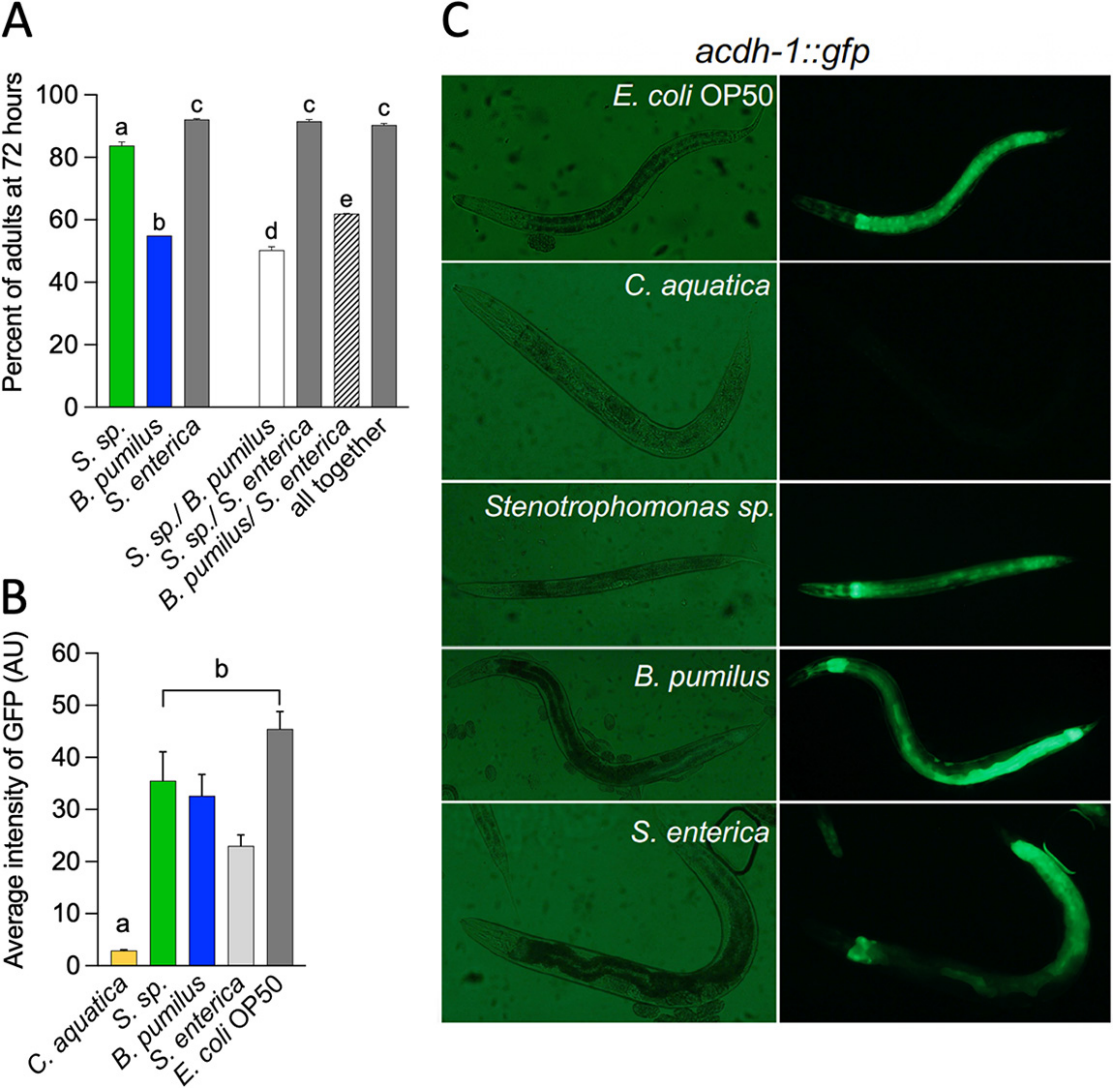
Development of animals in individual bacteria and consortia. (A) Percentages of animals in adult stages 72 h after exposure to single bacteria or consortia (*n* = 3) (one-way ANOVA). (B) Average intensity of *acdh-1*::*gfp* transgene expression in the intestine of C. elegans feeding on single bacteria (*n* = 3) (one-way ANOVA). AU, arbitrary units. (C) Representative images of the results from panel B. The same letter indicates no significant differences; different letters indicate significant differences. The underlying numerical data and statistical analysis for each figure panel can be found in S1 and S2 Datasets, respectively.

We measured the developmental rate of C. elegans feeding on the isolates. Animals were fed with individual and cocultures of bacteria, and the numbers of nematodes in each larval and adult stage were quantified every 24 h for 3 days. In the first 48 h, most animals developed similarly regardless of the diet (Fig. S2). At 72 h, a delay in growth was observed on *B. pumilus* alone and mixed with either *Stenotrophomonas* sp. or S. enterica compared with growth on other mixes also containing these two bacteria (Fig. 4A and B). Because the rate of pharyngeal pumping is high on *B. pumilus* (Fig. 3A), it is unlikely that this is due to low ingestion. An alternative is that *B. pumilus* lacks metabolites that accelerate development, such as vitamin B_12_ (20). Vitamin B_12_ can be obtained by bacteria by the *de novo* and/or the salvage B_12_ biosynthetic pathway (21). A screening of the genomes of both isolates for the genes required by these pathways indicates that neither of the pathways is complete, with both bacteria conserving only the genes for the first steps of the salvage pathway. Moreover, C. elegans acyl-CoA dehydrogenase (*acdh-1*) transcript levels are low in the presence of vitamin B_12_-producing bacteria such as Comamonas aquatica (4) and high in the presence of E. coli OP50, which does not produce the vitamin. *acdh-1*::*gfp*-expressing animals fed with the isolates caused green fluorescent protein (GFP) fluorescence to be high in the intestine (Fig. 4C and D). This shows that animals feeding on both wild isolates have high levels of *acdh-1*, likely suggesting that these bacteria lack vitamin B_12_.

10.1128/mbio.03402-22.3 FIG S3 Principal-component (PC) score plot derived from protective *B. pumilus* (blue) and nonprotective *Stenotrophomonas* sp. (green) and the mixture of *B. pumilus*-*Stenotrophomonas* sp. (gray). Download FIG S3, TIF file, 2.5 MB. Copyright © 2023 Urquiza-Zurich et al. 2023 Urquiza-Zurich et al. https://creativecommons.org/licenses/by/4.0/ This content is distributed under the terms of the Creative Commons Attribution 4.0 International license.

### The neuroprotective effect of *B. pumilus* is lost when mixed with *Stenotrophomonas* sp.

**FIG 5:**
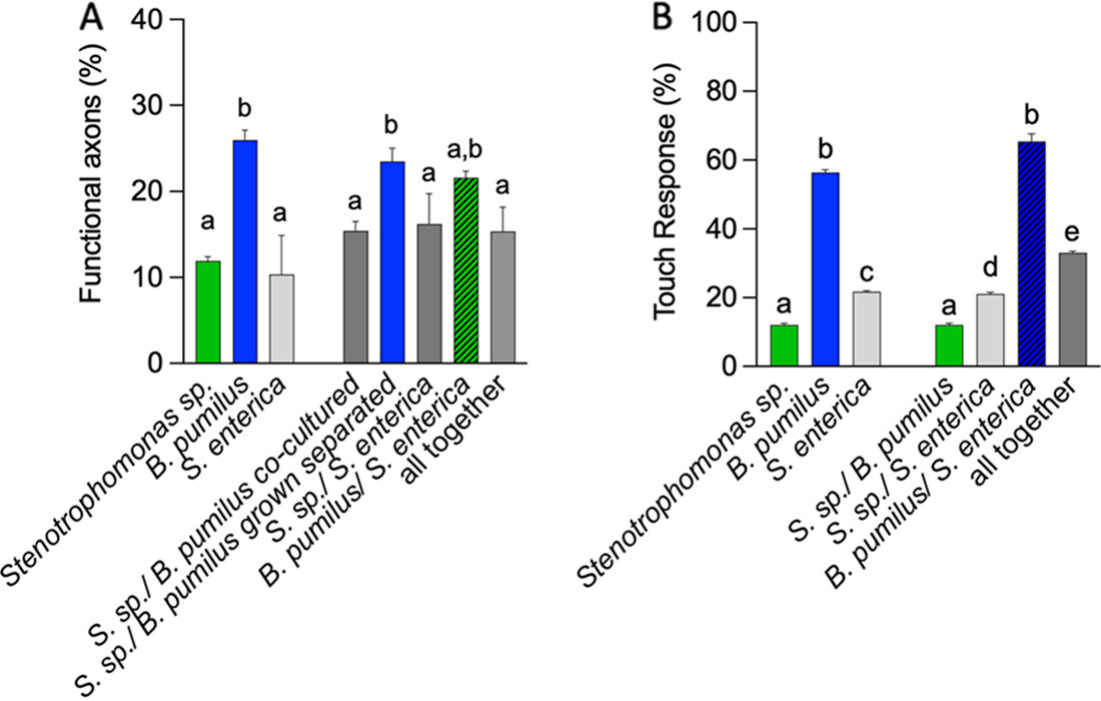
Neurodegeneration of the touch receptor neurons and response to touch in individual bacteria and consortia. Shown are the percentages of functional (protected) AVM axons (A) and touch responses (B) of animals feeding on single bacteria or consortia for one generation. *Stenotrophomonas* sp. and *B. pumilus* were fed to the worms either after coculture overnight or after being individually grown (separated). In both cases, animals were fed mixes of the bacteria. The same letter indicates no significant differences; different letters indicate significant differences. The underlying numerical data and statistical analysis for each figure panel can be found in S1 and S2 Datasets, respectively.

In C. elegans, GABA-producing bacteria prevent the genetically induced neurodegeneration of the touch receptor neurons (TRNs) (5). The TRNs are responsible for sensing gentle touch in the animal (22), a response that depends on the function of MEC-4 (23). A mutation that affects the extracellular domain of this protein (Val713) renders a channel defective in gating, promoting the unregulated influx of sodium and leading to the progressive degeneration of the cell (24). In the standard E. coli OP50 diet, the degeneration of the TRNs is complete 72 h after hatching, when animals are adults (25). In these worms, termed *mec-4d*, we tested whether the wild bacterial isolates and their consortia offer protection against the constitutive neurodegeneration of the anterior ventral microtubule (AVM) touch neuron (25). *B. pumilus* induced neuroprotection compared to the E. coli OP50 diet, while *Stenotrophomonas* sp. and S. enterica did not provide neuroprotection (Fig. 5A). AVM neuronal integrity conferred by the different bacterial diets correlated with the response to the gentle-touch stimulus (Fig. 5B). The mix of the two wild isolates eliminated the protection provided by *B. pumilus*, while the mix of *B. pumilus* with S. enterica did not (Fig. 5). These results suggest that the *B. pumilus* strain may produce metabolites with neuroprotective potential and that the expression and/or the stability of such metabolites is affected by its coculture with *Stenotrophomonas* sp. Neither of the genomes of the isolates conserved the genes for the glutamate dehydrogenase enzyme required for the biosynthesis of GABA, indicating that this may not be the metabolite responsible for neuroprotection in these bacteria.

### Metabolite profiling reveals NAD^+^ as a neuroprotector produced by native dietary bacteria.

To pinpoint which bacterial metabolites underlie the neuronal protection provided by *B. pumilus*, we extracted and analyzed the global metabolite contents of the isolates and their mixes by nuclear magnetic resonance (NMR) and multivariate data analyses.

**FIG 6:**
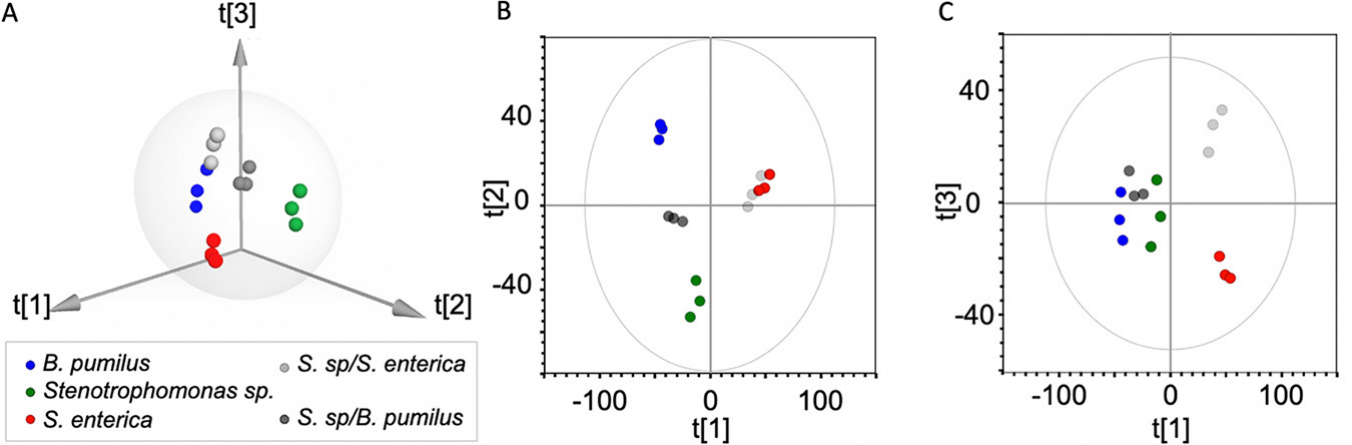
NMR metabolomics of bacterial extracts: exploratory data analysis by principal component (PC) analysis (PCA). PC score plots derived from ^1^H NMR spectra indicate the clustering of bacterial strains and their mixtures. (A) Three-dimensional (3D) score plot. (B and C) 2D score plot showing the first and second components (B) and the first and third ones (C).

We first identified the patterns and trends of clustering between samples by principal-component (PC) analysis (PCA) of binned ^1^H NMR data sets. In the PCA score plot (Fig. 6A), all samples fell within Hotelling’s *T*^2^ ellipse at 95% confidence intervals. Three PCs explain 77.3% of the data variation. The main order of variation was found in PC1, which explained 45.5% of the total variation and was associated with metabolic differences in S. enterica relative to *B. pumilus* and *Stenotrophomonas* sp. PC2 (22.1%) was associated with a pattern of variation between *Stenotrophomonas* sp. and *B. pumilus*, while PC3 (9.7%) denotes different metabolic profiles between S. enterica and the mixture of S. enterica with *B. pumilus* (Fig. 6B and C). Overall, the PCA revealed that the five groups of samples displayed different metabolic profiles, with S. enterica and the mixture of S. enterica with *Stenotrophomonas* sp. being the most distinct.

The neuroprotection provided by *B. pumilus* is lost when the bacterium is cocultured with *Stenotrophomonas* sp. (Fig. 5). To identify neuroprotective metabolites, we performed multivariate data analysis using the data for *Stenotrophomonas* sp., *B. pumilus*, and their mixture. PCA was used to explore the variation within each extract. Two PCs explained 78.1% of the total variation. The split samples from *Stenotrophomonas* sp. and *B. pumilus* were found in the PC1 (51.1%) direction, with the mixture of both being located in the middle. Metabolic variation due to bacterial coculture could be explained by PC2 (27%) (Fig. S3).

10.1128/mbio.03402-22.4 FIG S4 Validation of the OPLS-DA model of protective *B. pumilus* and nonprotective *Stenotrophomonas* sp. and the mixture of *B. pumilus*-*Stenotrophomonas* sp. by 200 permutations. Download FIG S4, TIF file, 2.5 MB. Copyright © 2023 Urquiza-Zurich et al. 2023 Urquiza-Zurich et al. https://creativecommons.org/licenses/by/4.0/ This content is distributed under the terms of the Creative Commons Attribution 4.0 International license.

**FIG 7:**
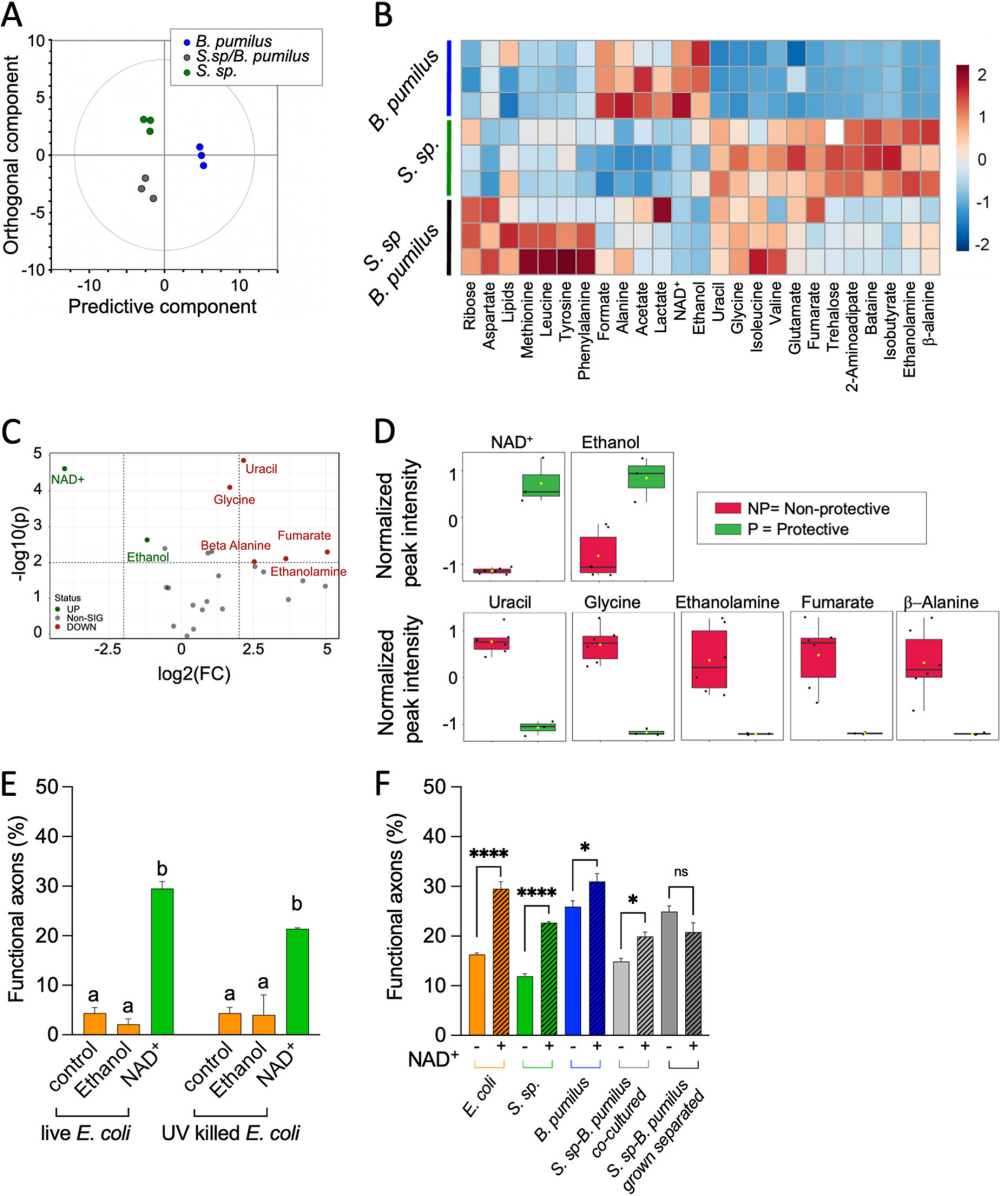
Metabolomics analysis of neuroprotective and nonprotective bacteria. The peak-intensity data matrix was normalized by probabilistic quotient normalization (PQN), mean centered, and autoscaled. (A) OPLS-DA score plot of protective *B. pumilus* and nonprotective *Stenotrophomonas* sp. and the mixture of *B. pumilus* and *Stenotrophomonas* sp. (B) Heat map showing differences in relative concentrations among the bacterial groups. The columns indicate different metabolites, whereas the rows indicate different samples. (C and D) Univariate metabolomics analysis. (C) Volcano plot analysis of metabolic changes in protective and nonprotective bacteria. Each point on the volcano plot was based on both *P* values and fold change (FC) values. The points that satisfy the conditions of a *P* value of <0.05 and a fold change of >2.0 appear in red (higher levels in protective bacteria) and blue (nonprotective bacteria). Nonsignificant (Non-SIG) markers appear in gray. (D) Box plots showing the normalized relative concentrations of significant metabolites selected in the volcano plot. (E) Percentages of functional AVM axons in *mec-4d* animals exposed to live and UV-killed E. coli OP50 supplemented with ethanol at 10 mM or NAD^+^ at 1 mM (*n* = 3) (one-way ANOVA). (F) Percentages of functional AVM axons in *mec-4d* animals exposed to wild isolates and their mixes supplemented with NAD^+^ at 1 mM. *Stenotrophomonas* sp. and *B. pumilus* were fed to the worms either after coculture overnight or after being individually grown (separated). In both cases, animals were fed mixes of the bacteria (*n* = 3) (one-way ANOVA). The same letter indicates no significant differences; different letters indicate significant differences. ****, *P* < 0.0001; ***, *P* < 0.001; **, *P* < 0.005; *, *P* < 0.05; ns, not significant. The underlying numerical data and statistical analysis for each figure panel can be found in S1 and S2 Datasets, respectively.

The orthogonal partial least-squares discriminant analysis (OPLS-DA) method (26) was used to search for discriminant metabolites observed in *B. pumilus* compared to *Stenotrophomonas* sp. and *Stenotrophomonas* sp. plus *B. pumilus*. The OPLS-DA score plot (Fig. 7A) shows intergroup metabolic differences. The discriminant supervised model was validated by 200 permutations (Fig. S4). A heat map was constructed to visualize low- and high-abundance metabolites for each bacterial extract and different groups (Fig. 7B). With the methodology used, ribose and aspartate were detected only in the coculture, while formate, alanine, NAD^+^, and ethanol were overrepresented in *B. pumilus* (Fig. 7B). Analysis of the variable importance in projection (VIP) plot reveals differences in metabolite levels between neuroprotective and nonprotective bacteria (Fig. S6). The metabolites with the highest VIP scores were NAD^+^, uracil, glycine, and ethanol.

10.1128/mbio.03402-22.5 FIG S5 Variable importance in projection (VIP) plot displaying the top 15 most important metabolite features identified by OPLS-DA. Colored boxes on the right indicate the relative concentration of the corresponding metabolite for samples from protective (P) and nonprotective (NP) bacteria. Download FIG S5, TIF file, 2.9 MB. Copyright © 2023 Urquiza-Zurich et al. 2023 Urquiza-Zurich et al. https://creativecommons.org/licenses/by/4.0/ This content is distributed under the terms of the Creative Commons Attribution 4.0 International license.

10.1128/mbio.03402-22.6 FIG S6 Percentage of functional AVM axons in *mec-4d* animals exposed to live (A) and UV-killed (B) E. coli OP50 supplemented with ethanol at 1, 10, 100, and 500 mM (*n* = 3) (one-way ANOVA). Download FIG S6, TIF file, 2.5 MB. Copyright © 2023 Urquiza-Zurich et al. 2023 Urquiza-Zurich et al. https://creativecommons.org/licenses/by/4.0/ This content is distributed under the terms of the Creative Commons Attribution 4.0 International license.

To focus on the independent changes in metabolite levels, univariate statistical analysis was employed to detect with higher stringency significant differences among the metabolome profiles of the groups. A volcano plot was used to select high-magnitude changes (fold change of >2.0) that are also statistically significant (*P* < 0.01) (Fig. 7C). This one-factor statistical analysis highlighted 7 differential metabolites (5 down- and 2 upregulated) in *B. pumilus* versus *Stenotrophomonas* sp. and *Stenotrophomonas* sp. plus *B. pumilus*. Univariate analysis revealed ethanolamine, β-alanine, glycine, uracil, and formic acid as being overrepresented under neurodegenerative conditions, whereas NAD^+^ and ethanol were overabundant in neuroprotective bacteria (Fig. 7C and D and Fig. S5). We do not expect all metabolites overrepresented in a neuroprotective bacterium to be necessarily neuroprotective. While it is unlikely that ethanol is neuroprotective, we selected both ethanol and NAD^+^ for *in vivo* testing of their effects on neuronal integrity. Ethanol has been shown to affect the behavior of C. elegans (27) in a concentration-dependent manner (28). Because bacteria can potentially metabolize supplements that have been added to their growth cultures, we tested both ethanol and NAD^+^ on live and UV-killed bacteria. As expected, ethanol did not improve the number of functional axons compared to control bacteria, either dead or alive (Fig. 7E and Fig. S6). On the contrary, NAD^+^ increased neuroprotection in live and UV-killed bacteria, indicating that NAD^+^ is neuroprotective (Fig. 7E).

We next added NAD^+^ to different assortments of bacteria and fed them to *mec-4d* animals. We observed neuroprotection in worms grown on E. coli OP50, *Stenotrophomonas* sp., and *B. pumilus* supplemented with NAD^+^ (Fig. 7F), suggesting that NAD^+^ neuroprotection is independent of the diet and other metabolites being synthesized. The addition of NAD^+^ to the coculture of *Stenotrophomonas* sp. and *B. pumilus* also increased the protection of *mec-4d* TRNs. When both bacteria were grown separately and then fed to the worms, the neuroprotection of *B. pumilus* was not affected, and the addition of NAD^+^ did not increase neuroprotection (Fig. 7F).

## DISCUSSION

Microbivore nematodes are a fundamental component of the biosphere (29), and their fitness as a species is used as a parameter to test soil and niche health (30). The microbiota and microbial relationships that animals establish are likely to impact the behavior of soil nematodes and therefore are tightly linked to the health of these niches. Here, we quantified how life-history traits change as a consequence of feeding on *Stenotrophomonas* sp. and *B. pumilus*, which were coisolated with wild nematodes, and tested how feeding on their cocultures affected worm life-history traits and behavioral outputs. From our findings on the loss of neuroprotection in worms fed on *B. pumilus* cocultured with *Stenotrophomonas* sp., we identified and validated *in vivo* the neuroprotective properties of NAD^+^ on C. elegans’ touch circuit. The fact that the mix of the two isolates is neurodegenerative only when the isolates are cultured together and not when they are given together to the nematodes from independent cultures indicates that the growth dynamics of the bacterial coculture affect the host’s life-history traits.

### Effect of isolates and their consortia on life-history traits.

*Stenotrophomonas* and *Bacillus* isolated with wild South American bacterivore nematodes have also been found as part of the microbiota of nematodes elsewhere (6, 7, 31), thus likely making the association of these bacteria with wild worms not incidental. However, whether these isolates comprise a preferred diet in the context of a natural environment or are part of the nematode microbiota needs further assessment. Worms may be able to sense and prefer environmental bacteria that are advantageous. For example, a natural isolate of *Pantoea* that accelerates development and protects against pathogen colonization is better fitted to colonize the worm and is consistently selected as the diet over other environmental strains (32).

Wild animals strongly preferred the isolates over E. coli OP50. C. elegans slightly preferred its regular E. coli diet, at least for the first generation, which is consistent with animals preferring what is familiar to them (16). However, when offered alone, C. elegans remained in lawns with the isolates, suggesting that in the absence of familiar smells and tastes, it senses natural bacteria as a beneficial diet. The molecular mediators of this behavioral response were not explored, but it is likely that metabolites produced by the bacteria are responsible for the attraction or repulsion of the nematodes. For example, C. elegans may regulate its food intake level depending on the nutritional quality of the diet by sensing its content of vitamin B_2_ (33). Tyramine produced by an environmental *Providencia* isolate directly affects the olfactory choices of the worm, making it prone to keep selecting *Providencia* as food (34).

The acceleration of reproductive age together with progeny numbers are considered strong indicators of fitness (35). Interestingly, C. elegans growing in a mix of the two isolates delays development compared to any other diet, thus suggesting that in nature, as animals feed on a mixture of different bacterial species, the growth dynamics that they experience may be slower, perhaps contributing to longevity in the wild (36).

Food availability directly modulates the rate of pharyngeal pumping (37). However, the rates of pharyngeal pumping are similar in bacteria that worms can and cannot eat (17, 38), suggesting that the pumping rate alone is not sufficient to measure food intake. We find that in both monoxenic cultures and mixes of *B. pumilus* with other bacteria, the pharyngeal pumping rate increased, even when it was mixed with *Stenotrophomonas* sp., which by itself causes the lowest pumping rate. This may also imply that bacteria that are more difficult to ingest can induce an increase in pumping to compensate for the low food load in the digestive tract. Furthermore, we cannot rule out whether *B. pumilus* produces a molecule or metabolite that generates an increase in pharyngeal pumping (39, 40).

### Bacterial components and neurodegeneration of the touch circuit.

In the wild, nematodes use the touch circuit to escape predators such as nematophagous fungi (41). *B. pumilus* protects the neurons of C. elegans from constitutive genetically induced neurodegeneration by producing the metabolite NAD^+^, which is involved in preventing oxidative stress and DNA damage in vertebrates (42, 43). We previously showed that the overexpression of NMAT-2, the NAD^+^-producing enzyme, protects TRNs from *mec-4d*-induced neurodegeneration (25). In this work, we show that NAD^+^ produced by wild bacteria is relevant for the neuroprotection of the host and that this protection is lost when *B. pumilus* is cocultured with *Stenotrophomonas* sp. While we previously found metabolites from neuroprotective laboratory E. coli (5), this work highlights the need to extend the search for novel neuroprotective molecules in bacteria outside laboratory settings.

Animals fed with a combination of two or three bacteria, independently of their origin or nutritional value, changed their phenotypic outcomes dramatically in comparison with feeding on individually grown bacteria. This highlights that the understanding of the effect of the microbiota on animal and human physiology requires the study of complex bacterial associations.

## MATERIALS AND METHODS

### Isolation of native worms and coexisting bacteria.

Samples were obtained from soils presupplemented with fruits that rot *in situ*. Fresh, washed apples were cut and buried in the soil under a native Schinus polygama tree surrounded by native Acacia caven trees (33°22′38.3″S, 70°36′54.0″W). Two weeks later, a teaspoon of soil was collected, along with the decomposed apple remains. The sample was divided and deposited into three 60-mm petri dishes containing nematode growth medium (NGM) with small lawns of E. coli OP50 (1 cm in diameter). Plates were checked every 12 h, and once the nematodes appeared, they were transferred to new NGM plates with fresh E. coli lawns. The plates with the wild nematodes grew bacterial colonies on their E. coli-free surface, allowing their isolation. Bacterial colonies that contained worms feeding on them were isolated in lysogeny broth (LB) and NGM plates at 25°C. Because we cannot be certain that these colonies are nematode associated in the wild, we name them coisolated to indicate that they came from the same soil sample and were carried by worms to the new plates.

### C. elegans maintenance and growth.

Wild-type N2 C. elegans, *mec-4d* mutants, and the transgenic strain VL749 [*acdh-1p*::GFP + unc-119(+)] were grown at 20°C as previously described (3). All strains were maintained on E. coli OP50 prior to feeding with other bacteria. All life-history traits were quantified starting with synchronized animals.

### Nematode synchronization.

Larvae and adults were removed from plates full of laid embryos using M9 buffer. Embryos remained attached to the plates and were allowed to hatch for 2 h. Zero to two hours after hatching, L1 larvae (first larval stage) were picked with a mouth pipette into M9 buffer and placed onto experimental NGM plates seeded with the desired bacteria.

### Bacterial growth.

The following bacteria were used as C. elegans diets: E. coli OP50, E. coli HT115, S. enterica serovar Typhimurium, C. aquatica, *Stenotrophomonas* sp., and *B. pumilus*. Bacteria were grown overnight on LB plates at 30°C (*Stenotrophomonas* sp. and *B. pumilus*) and 37°C (E. coli and S. enterica) from frozen glycerol stocks. The next morning, a scoop of the bacterial lawn was inoculated into LB with antibiotics when required. Streptomycin at 25 μg/mL (Calbiochem) was used for E. coli OP50, and cultures were grown for 6 h with agitation at 200 rpm at 37°C (until an optical density at 600 nm [OD_600_] of 1.5 to 2.0 was reached). A 100-μL volume of the bacterial cultures was seeded onto 60-mm NGM plates and allowed to dry overnight.

### Bacterial growth curve.

An initial 3-mL preinoculum of each bacterium was grown in a 15-mL Falcon tube in liquid LB without antibiotics at 30°C in a Lab Companion SI-300R refrigerated benchtop shaker at 200 rpm for 14 to 16 h. This culture was then grown in 10 mL of LB in a 50-mL Falcon tube as explained above. This culture was then diluted to reach an OD of 0.1 nm as measured using a BK-UV1900/BK-V1900 scanning UV-visible (UV-Vis) spectrophotometer at 600 nm to obtain an initial culture.

Hourly bacterial growth was quantified in cultures in Nunc MicroWell 96-well plates (Thermo Fisher Scientific), which were read with a Nanoquant Tecan Infinite 200Pro system. Fifty microliters of each bacterium culture was placed into a well, which was filled with 150 μL of liquid LB. The second row was used as a control, containing 200 μL of LB. Plates were cultures with orbital agitation at 200 rpm at 30°C, and the OD was measured every hour for 18 h.

### UV killing of bacteria.

E. coli OP50 bacteria were grown overnight in LB with agitation (450 × *g*) at 37°C. The next morning, 5 mL of the culture was placed onto empty sterile 90-mm plates and exposed to UV light (302 nm) for 15 min in a UV transilluminator (MaestroGen). Samples of the treated bacteria were confirmed to be killed by allowing them to grow overnight on LB plates at 37°C.

### NAD^+^ and ethanol supplementation.

A final concentration of 1 mM NAD^+^ (Sigma) or ethanol (1, 10, 100, and 500 mM, 96%; Supelco) was added to live or UV-killed liquid bacteria before seeding them onto NGM plates. *mec-4d* embryos obtained from the synchronization of gravid adults were placed onto the bacterial lawn 8 to 12 h after they were seeded.

### Quantification of life-history traits.

**(i) Developmental rate.** Sixty-millimeter NGM plates were seeded with 100 μL of the desired bacteria previously cultured to an OD_600_ of 0.4. Thirty to forty L1 worms at 0 to 2 h posthatching were placed onto each plate. Every 24 h for 3 days, the number of animals in each developmental stage was counted under a Nikon SMZ745T stereomicroscope with a Nikon G-AL 2× objective.

**(ii) Pharyngeal pumping.** Thirty adult animals (12 h after L4) were observed under a stereomicroscope at high magnification. The number of pumps per minute of the animals was quantified using a manual counter.

**(iii) Defecation intervals.** The time that elapsed between defecation events in 30 adult animals (12 h after L4) observed under a stereomicroscope at high magnification was recorded using a chronometer.

**(iv) Locomotion pattern.** Ten to fifteen adult nematodes were placed inside a bacterial lawn. Worm tracks were observed after 1 h and thereafter monitored for one more hour. If nematodes displayed movements with recurrent stops and turns, this was recorded as dwelling. If they showed rapid and incessant movement, this was recorded as roaming (17).

**(v) Bacterial preference.** Food choice experiments with single and mixed isolates were done in comparison with the control bacteria E. coli OP50 and E. coli HT115. Sixty-millimeter NGM plates were prepared without antibiotics and inoculated with bacteria at an absorbance of 0.4 nm on each side of the plate, generating a lawn of approximately 1 cm in diameter (30 μL). The plates were then left for 24 h at room temperature to dry, and the next day, 30 worms in stage L4 previously fed with E. coli OP50 were placed at the border of the plate equidistant from each bacterial lawn. Animals were placed on an antibiotic-free NGM plate for 30 min to clean them of bacteria before placing them onto the plates of preference. After being placed onto the plate, the location of animals was quantified at 30 min and 1, 2, 3, 4, 12, and 24 h. The chemotaxis index was calculated using the formula (no. of worms on test bacteria − no. of worms on the E. coli control)/total no. of animals.

**(vi) Distribution in plates.** Sixty-millimeter NGM plates were seeded with 300 μL of a bacterial inoculum and incubated at 37°C with agitation until an OD of 0.4 nm was reached. The next day, 30 L4 animals previously fed on E. coli OP50 were placed onto an antibiotic-free NGM plate to clean them of bacteria before being transferred by picking and placed in the center of the lawn. The numbers of worms inside and outside the lawn were quantified at 1, 2, and 3 h.

**(vii) Neuronal integrity.** Neurons with full-length axons were classified as AxW. Neurons with axons with a process connected to the nerve ring were classified as AxL, and those with axons that did not reach the bifurcation to the nerve ring were classified as AxT. The absence of the neuron, or neurons presenting only the soma, as well as neurons with axons conserving only the ventral projection were classified as Axϕ.

**(viii) Touch response.** Animals were gently touched with an eyelash (22) in the anterior area over the second bulb of the pharynx. A positive response was recorded when animals moved backward as a result of being touched with the eyelash.

### Quantification of *acdh-1*::*gfp* expression.

Animals were mounted onto 200-μm-thick agarose pads with 1 mM levamisole and visualized on an upright Eclipse Ti microscope (Nikon Instruments) with a 40 × /0.75-numerical-aperture (NA) objective, and images were acquired with an Eos rebel t3i camera (Canon). Fluorescence was quantified using ImageJ software by cropping the region of interest (ROI) corresponding to individual animals and taking the average intensity of pixels within each ROI.

### Statistical evaluation and experimental size.

All experiments were done at least 3 times (3 biological replicates, started on different days and from different starting plates). Each biological replicate contained a triplicate (three technical replicates). Statistical evaluation was done by one- or two-way analysis of variance (ANOVA) with *post hoc* analyses.

### Genomics.

**(i) Extraction of bacterial DNA.** Genomic DNA was extracted from cultures grown to an OD_600_ of between 0.4 and 0.6 nm using the Ultra Clean microbial DNA isolation kit (MoBio Laboratories) according to the instructions of the manufacturer. Genomic DNA samples were quantified on a Nanoquant Tecan Infinite 200Pro instrument. Genomic DNA samples from the bacterial isolates were treated with RNase A (Qiagen) for 60 min at 37°C to eliminate RNA remnants from the samples.

**(ii) Genome sequencing and identification of bacteria.** The genomic DNA samples of each bacterial isolate were sequenced at Genoma Mayor SpA, employing the TruSeq Nano DNA LT kit (Illumina). A quality control of the sequenced reads of each sequenced sample was performed using the FastQC program. Next, reads were assembled using SPAdes. The quality of the genome assemblies was assessed with Quast (http://quast.bioinf.spbau.ru/). Genome assemblies were annotated using the RAST toolkit from PATRIC. To identify the bacteria, genome sequence contigs were uploaded to the Type Strain Genome Server (TYGS) (https://tygs.dsmz.de/) for taxonomy-based identification analysis. The resulting 16S rRNA-based and genome BLAST distance phylogeny (GBDP)-based phylogenetic trees and the digital DNA-DNA hybridization result table were retrieved. From this analysis, the closest type strain to each isolate was determined. Next, the genome sequences of the closest type strains were retrieved from GenBank and used to determine the average nucleotide identity (ANI) between the isolates’ genomes and their respective closest type strains using OrthoANIu (https://www.ezbiocloud.net/tools/ani).

### Metabolomics.

**(i) Generation of bacterial extracts.** Bacteria were cultured in 2 mL of liquid LB at 30°C at 200 rpm overnight. The preincubated inoculum was placed into 30 mL of liquid LB in 50-mL Falcon tubes and grown at 30°C at 200 rpm until an OD_600_ of 1 was reached. Bacterial cultures were centrifuged at 6,500 × *g* for 5 min, an aliquot of 1 mL of the supernatant was stored at −20°C, and the rest was discarded. The bacterial pellet was resuspended in 20 mL of a cold phosphate-buffered saline (PBS) solution (NaCl [137 mM], KCl [2.7 mM], Na_2_HPO_4_ [10 mM], and KH_2_PO_4_ [1.8 mM]). Pellets were centrifuged again and washed twice with 1 mL of cold PBS. After the washes, the pellet was stored at −20°C. The pellet was resuspended in 600 μL of extraction buffer (acetonitrile and KH_2_PO_4_-NaH_2_PO_4_ at 100 mM [pH 7.4]) and mixed by vortexing for 30 s. Next, the tubes containing the resuspended pellet were placed into liquid nitrogen for 1 min and then on ice until they were thawed (4°C). The vortexing and freezing in liquid nitrogen steps were repeated two more times, and the tubes were then sonicated in an ultrasonic water bath (Bioruptor UCD-200; Diagenode) for 15 cycles of 30 s on and 30 s off at maximum power. Next, the samples were centrifuged at 12,000 rpm for 10 min, and the supernatants were recovered in new sterile tubes. The remaining pellet was extracted again as described above, and the supernatant resulting from the second extraction was added to the first supernatant to optimize the recovery of metabolites. The supernatants were dried in a vacuum dryer (Savant) at 50°C for 2 to 3 h depending on the number of samples. Once the samples were dried, they were stored at −20°C until metabolic profiling.

**(ii) Sample preparation for ^1^H NMR spectroscopy.** ^1^H NMR spectroscopy and multivariate data analyses were performed at the Platform for Structural and Metabolomic Biology (PLABEM), Rosario, Santa Fe, Argentina. Samples were randomized and reconstituted in 600 μL of 100 mM Na^+^/K^+^ buffer (pH 7.4) containing 0.005% sodium 3-trimethylsilyl-(2,2,3,3-2H4)-1-propionate (TSP) and 10% D_2_O. In order to remove any precipitate, samples were centrifuged for 10 min at 14,300 × *g* at 4°C. An aliquot of 500 μL of the centrifuged solution was transferred to a 5-mm NMR tube (Wilmad LabGlass).

**(iii) ^1^H NMR spectroscopic analysis of bacterial extracts.** Water-suppressed ^1^H NMR spectroscopy was performed at 300 K on a Bruker 700-MHz spectrometer equipped with a 5-mm triple resonance inverse (TXI) probe (Bruker Biospin, Rheinstetten, Germany) using a standard one-dimensional (1D) NOESY pulse sequence with presaturation and spoil gradients (noesygppr1d) (44, 45). The ^1^H NMR spectra were acquired using 4 dummy scans, 32 scans with 64,000 time domain points, and a spectral window of 20 ppm. The mixing time was set to 10 ms, the data acquisition period was set to 2.228 s, and the relaxation delay was set to 4 s. The free induction decays (FIDs) were multiplied by an exponential weighting function corresponding to a line broadening of 0.3 Hz.

**(iv) ^1^H NMR spectral processing.** NMR spectra were processed in Matlab (version R2016b; The MathWorks). Spectra were referenced to TSP at 0.0 ppm, and baseline correction and phasing of the spectra were achieved using Imperial College written functions (provided by T. Ebbels and H. Keun, Imperial College London). Each spectrum was divided into integrated regions of equal widths (0.04 ppm, bucket width). Noninformative spectral regions containing TSP and water signals were excluded. Each spectrum was then normalized by the probabilistic quotient method (46). Spectral alignments were made using the recursive segment-wise peak alignment algorithm (47) in user-defined windows, and the full spectral matrix was exported.

**(v) NMR resonance integration.** The resolution and integration of all resonances of the spectral data set were performed using the multivariate curve resolution-alternating least-squares (MCR-ALS) method as proposed previously by Pérez et al. (48). Briefly, the spectral mode was manually segmented into 63 spectral subregions that were then analyzed using MCR-ALS. From the 63 integrated regions, 25 metabolites were assigned using public NMR databases (49), whereas 35 resolved curves could not be assigned. Unknown features were discarded, and assigned features were selected to build the final matrix. In cases where more than one signal from a compound was integrated, the better-resolved and less-overlapped one was selected to build the matrix. The description of integrated regions and the number of considered components are detailed in Table S1 in the supplemental material. In cases where more than one signal per molecule was considered, integrated values were averaged.

10.1128/mbio.03402-22.7 TABLE S1 Integrated regions and number of considered components. Download Table S1, XLSX file, 0.01 MB. Copyright © 2023 Urquiza-Zurich et al. 2023 Urquiza-Zurich et al. https://creativecommons.org/licenses/by/4.0/ This content is distributed under the terms of the Creative Commons Attribution 4.0 International license.

**(vi) Multivariate data analysis.** The full spectral matrices corresponding to single and mixed bacteria were imported to SIMCA 16 (Umetrics AB, Umeå, Sweden). Principal-component analysis (PCA) was performed on the mean-centered and Pareto-scaled NMR data set, and score plots were analyzed. The absence of outliers was evaluated by Hotelling’s *T*^2^ ellipse at 95% confidence intervals.

To evaluate neuroprotection, an integrated peak matrix was imported into SIMCA. Data were mean centered and unit variance (UV) scaled. A PCA plot was built for global data visualization, and orthogonal partial least-squares discriminant analysis (OPLS-DA) was performed to maximize the separation between bacterial groups as a function of neuroprotection (26). To ensure valid and reliable OPLS-DA models and to avoid overfitting, 200 permutations were carried out. Discriminant features between classes in the OPLS-DA models were defined using a loading plot.

### One-factor statistical analysis.

The peak integral matrix was imported into MetaboAnalyst 5.0 (50). Volcano plots were used, with a fold change of 2.0 and a *P* value of <0.05.

### Data availability.

The genomic data on the bacteria identified in this study have been deposited in the BioProject PRJNA876939 under the accession JAODBT000000000 (*Stenotrophomonas* sp. Iso1) and JAODBS000000000 (*Bacillus pumilus* Iso2).

10.1128/mbio.03402-22.8 DATA SET S1 All data obtained in the manuscript. Each sheet refers to data for one figure. (Sheet 1) Data for Fig. 2; (sheet 2) data for Fig. 3; (sheet 3) data for Fig. 4; (sheet 4) data for Fig. 5; (sheet 5) data for Fig. 7; (sheet 6) data for Fig. S1 in the supplemental material; (sheet 7) data for Fig. S2; (sheet 8) data for Fig. S6. Download Data Set S1, XLSX file, 0.02 MB. Copyright © 2023 Urquiza-Zurich et al. 2023 Urquiza-Zurich et al. https://creativecommons.org/licenses/by/4.0/ This content is distributed under the terms of the Creative Commons Attribution 4.0 International license.

10.1128/mbio.03402-22.9 DATA SET S2 All statistics for the data in the manuscript. Each sheet refers to statistics for one figure. (Sheet 1) Statistics for Fig. 2; (sheet 2) statistics for Fig. 3; (sheet 3) statistics for Fig. 4; (sheet 4) statistics for Fig. 5; (sheet 5) statistics for Fig. 7; (sheet 6) statistics for Fig. S6 in the supplemental material. Download Data Set S2, XLSX file, 0.03 MB. Copyright © 2023 Urquiza-Zurich et al. 2023 Urquiza-Zurich et al. https://creativecommons.org/licenses/by/4.0/ This content is distributed under the terms of the Creative Commons Attribution 4.0 International license.
