# Supplementary figures and images for: The Assembly of Bacteria Living in Natural Environments Shapes Neuronal Integrity and Behavioral Outputs in Caenorhabditis elegans
Source: mBio. 2023 Mar 8;14(2):e03402-22. doi: 10.1128/mbio.03402-22 (PMC10127743; doi:10.1128/mbio.03402-22)

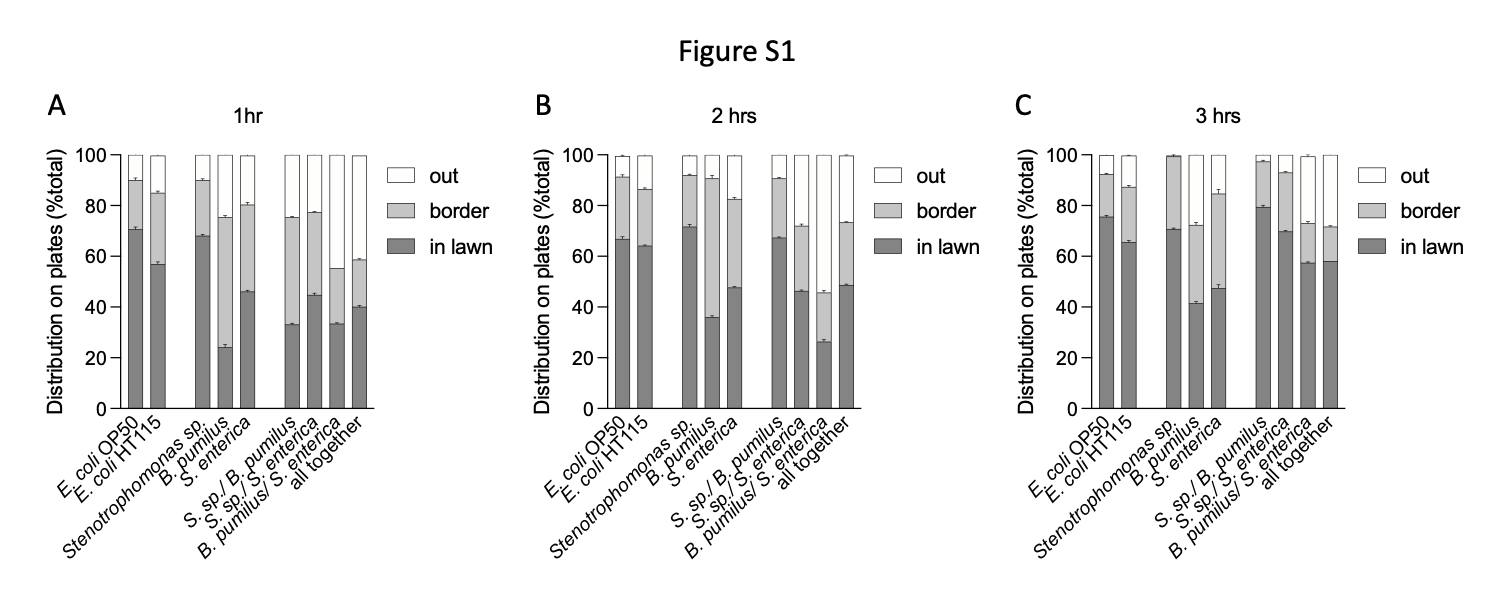

Supplement: FIG S1 [file mbio.03402-22-s0002.tif]

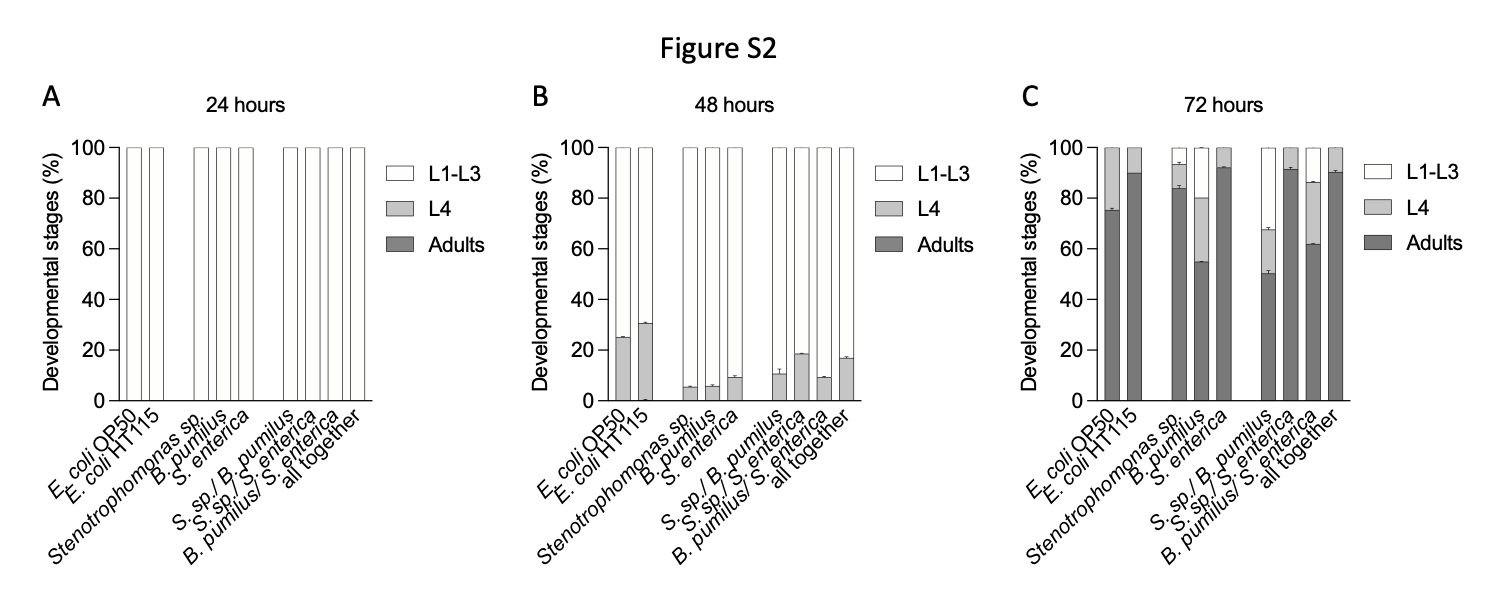

Supplement: FIG S2 [file mbio.03402-22-s0003.tif]

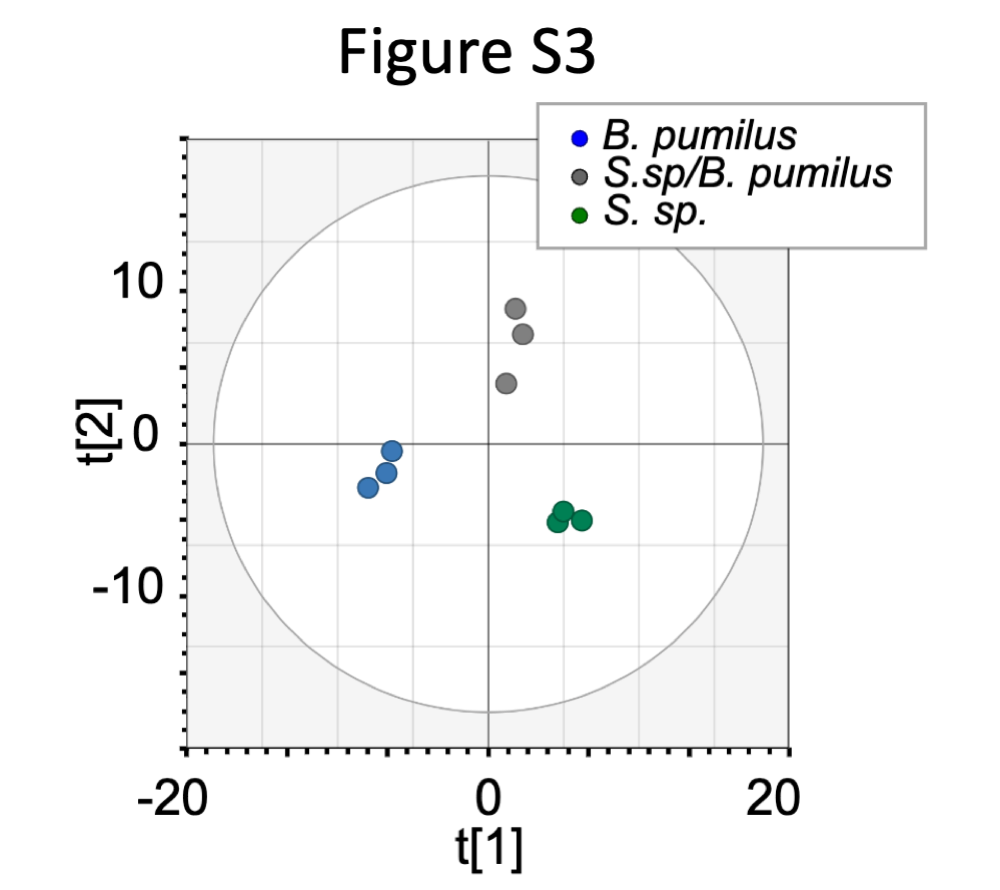

Supplement: FIG S3 [file mbio.03402-22-s0004.tif]

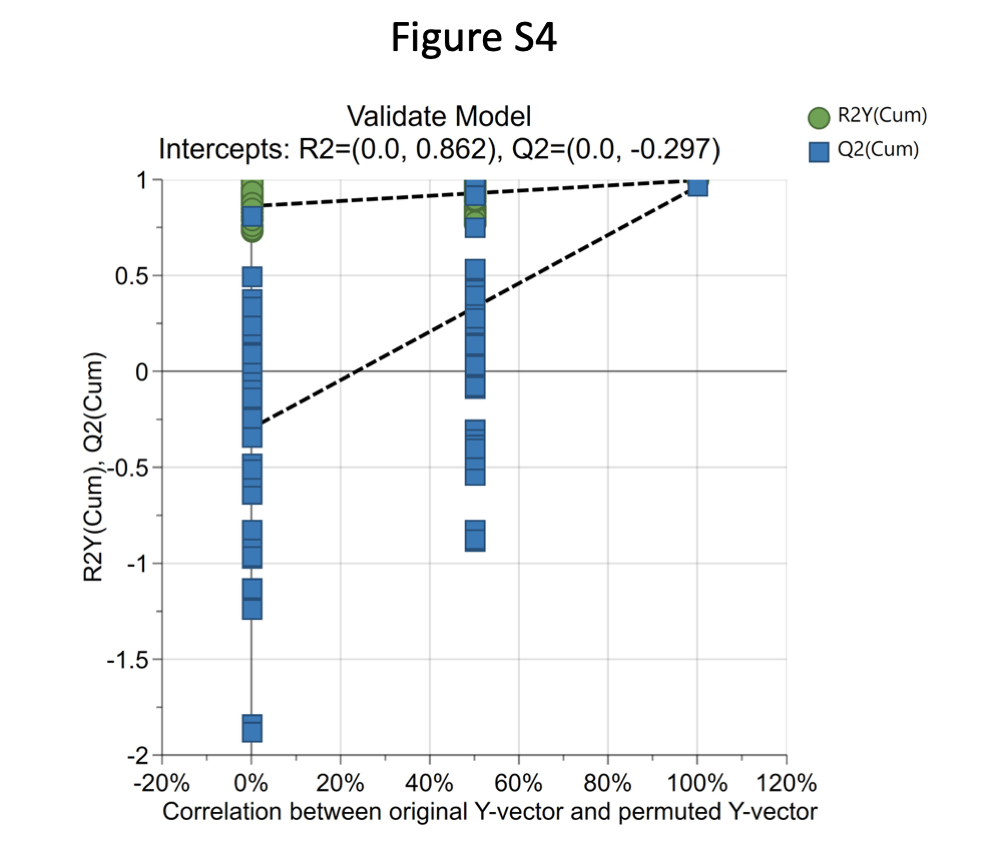

Supplement: FIG S4 [file mbio.03402-22-s0005.tif]

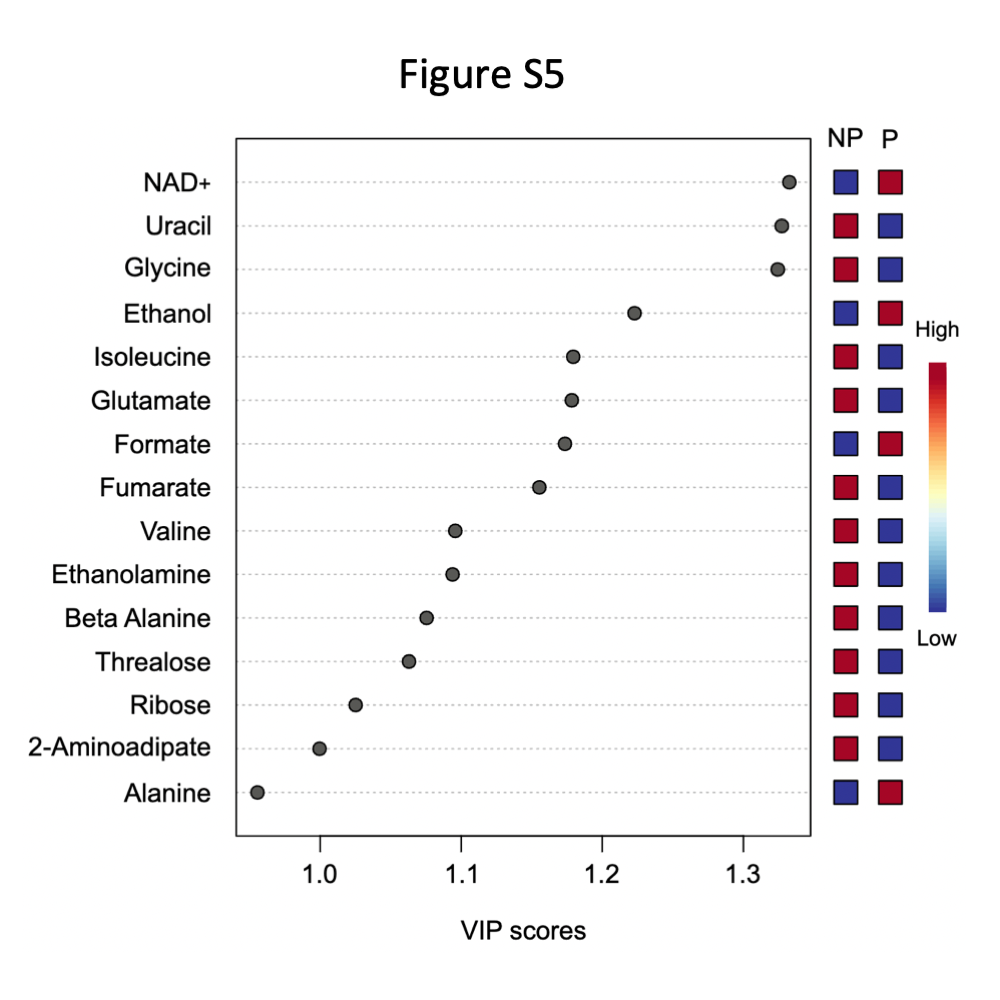

Supplement: FIG S5 [file mbio.03402-22-s0006.tif]

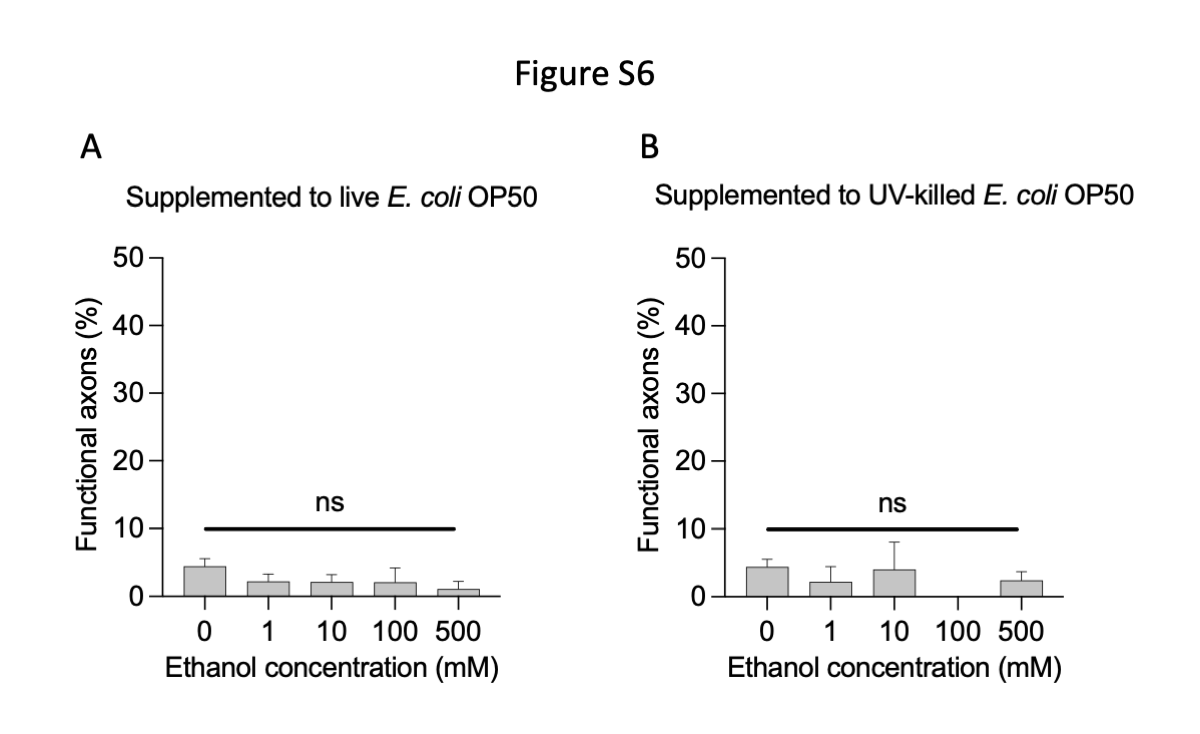

Supplement: FIG S6 [file mbio.03402-22-s0007.tif]
